# Mid-term outcomes of right subaxillary approach versus median sternotomy incision for ventricular septal defect with patent ductus arteriosus

**DOI:** 10.1186/s12887-022-03757-6

**Published:** 2022-11-29

**Authors:** Zhi-Huang Qiu, Qing-Song Wu, Jun Xiao, Tian-Ci Chai, Mi-Rong Tang, Xian-Biao Xie, Dong-Shao Liao, Liang-Wan Chen

**Affiliations:** 1 Department of Cardiovascular Surgery, Union Hospital, Fujian Medical University, Fuzhou, Fujian China; 2 Key Laboratory of Cardio-Thoracic Surgery Fujian Medical University, Fujian Province University, Fuzhou Fujian, China; 3 Fujian Provincial Special Reserve Talents Laboratory, Fuzhou Fujian, China

**Keywords:** Ventricular septal defect, Patent ductus arteriosus, Right subaxillary approach, Median sternotomy incision, HRQoL

## Abstract

**Background:**

This study aimed to evaluate and compare two surgical approaches to repair ventricular septal defect (VSD) with patent ductus arteriosus (PDA) and to explore the patients’ health-related quality of life (HRQoL).

**Methods:**

We conducted a retrospective study of all patients who had surgical repair of VSD and PDA between 2013 and 2015 using the right subaxillary approach (group A) or the median sternotomy incision (group B). The outcomes of both techniques were compared. Paediatric QoL Inventory 4.0 scale was applied to assess patients’ HRQoL in the 6th postoperative year. Multiple linear regression analysis was performed to explore factors associated with higher HRQoL scores.

**Results:**

A total of 128 patients were included (group A, *n* = 70 and group B, *n* = 58). Patients in group A were older and heavier than patients in group B. In group B, the diameters of VSD and PDA were larger and the pulmonary artery pressures were higher than those in group A (*p* < 0.001). No mortality occurred on a mean follow-up of 8.3 ± 1.2 years. Patients in group A had higher HRQoL scores than those in group B in terms of emotional and social functioning dimensions. The right subaxillary approach (OR: 3.56; 95% CI 1.65–5.46), higher parents’ education level (OR: 1.62; 95% CI 0.65–2.31), and better family economic status (OR: 1.48; 95% CI 0.79–2.45) were associated with higher HRQoL scores.

**Conclusions:**

Younger and smaller patients receiving median sternotomy incisions due to large defects and pulmonary hypertension had lower HRQoL scores. The right subaxillary approach, higher parents’ education level, and better family economic status were associated with higher HRQoL scores.

## Introduction

With the increasing number of patients demanding cosmetic incisions, surgeons continued to explore more techniques for concealed incisions, such as mini-sternotomy and anterolateral thoracotomy, to correct simple congenital heart disease (CHD) [1]. The right subaxillary approach concealed the incision better than other techniques and had been widely used in the correction of simple CHD. Many CHD patients, including those with ventricular septal defect (VSD), atrial septal defect, partial anomalous pulmonary venous connection, and partial atrioventricular septal defect, had undergone operation through a right subaxillary approach [2–6]. At present, many studies had reported that compared with the median incision, the right subaxillary incision for repairing simple CHD had the advantages of cosmesis and faster recovery [7, 8]. Hu and colleagues reported that the short-term and mid-term results of VSD closure for 429 children were satisfactory [7]. In a series of 136 cases, Hu and colleagues also reported the right subaxillary incision for common CHDs was as safe as median sternotomy. In addition, the former was better than the latter in terms of hidden incision, appearance, and postoperative recovery [8].

However, the long-term health-related quality of life (HRQoL) in children with CHD after surgery was a complex concept. There were many influencing factors including surgical scar, CHD severity, parents’ education level, and family economic status [9, 10]. This study aimed to evaluate the perioperative outcomes of repairing VSD with patent ductus arteriosus (PDA) through two different surgical approaches and to explore the patients’ HRQoL.

## Patients and methods

### Study design

This study included patients who underwent repair of VSD with PDA between January 2013 and January 2015. The patients were divided into two groups according to the surgical approach used: the right subaxillary approach (group A) and the median sternotomy incision approach (group B). In clinical practice, combining previous literature and the clinical experience of cardiac surgeons [6, 10, 11], patients who met the following inclusion criteria received the right subaxillary approach: age ≥ 6 months, weight ≥ 5 kg, body mass index < 30 kg/m^2^, no severe aortic regurgitation, no respiratory disease, and no history of a right thorax procedure. Other patients received the median sternotomy incision approach.

### Surgical technique

**Fig. 1:**
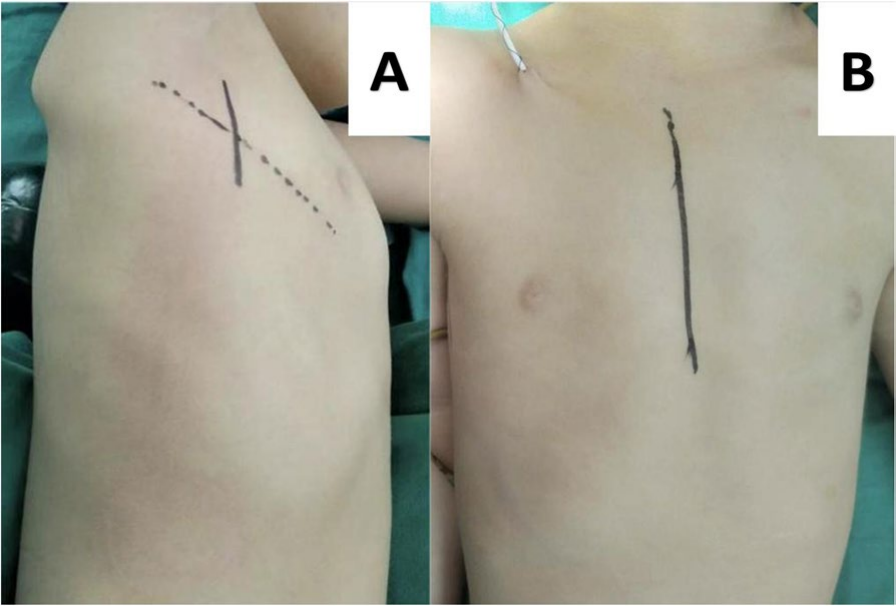
**A** The position of the patient when undergoing a right subaxillary approach. **B** The position of the patient when undergoing median sternotomy incision

The surgical technique for the right subaxillary approach was based on previous literature reports [2, 3]. The procedure involved placing the patient in a left lateral decubitus position (Fig. 1A), creating a 5–8 cm incision, and entering into the right thoracic cavity from the fourth intercostal space.

After cardiopulmonary bypass (CPB) was in place, the PDA was ligated or closed through an incision on the main pulmonary artery. Because of its deep location, the PDA could not be clearly exposed in the left lateral decubitus position, and it must be carefully separated. After cardioplegic perfusion, the right atrium or main pulmonary artery was opened to explore the defect according to the different types of VSD. The VSD was repaired with an autologous pericardial patch using a running suture with a 6–0 propylene suture. Then, the CPB was withdrawn gradually. After closing the chest, the muscle and skin incision were carefully sutured.

For the median sternotomy incision, the patients were placed in the supine position (Fig. 1B). After the CPB was in place, the PDA was first ligated or closed. The VSD closure was completed through different approaches based on the VSD type. The defect was also closed with an autologous pericardial patch using a running suture.

All patients underwent transoesophageal echocardiography during the operation to detect residual shunt and other anomalies.

### Follow-up

All patients were followed-up in the outpatient cardiac department. Echocardiography was performed at discharge, 3 and 6 months after surgery, and annually thereafter, to detect abnormal cardiac function, valve regurgitation, and residual shunt.

We used the Pediatric Quality of Life Inventory 4.0 (PedsQL 4.0) scale to assess the HRQoL of the patients. The HRQoL survey was conducted once in the 6th postoperative year for all patients. The research team consisted of one pediatric cardiac surgeon and two pediatric cardiac nurses. The same research team completed the questionnaires of the two groups. The researchers were allowed to help the subjects understand the questions, but they were not allowed to induce or interfere with the answers to ensure the authenticity of the questionnaire.

### HRQoL

PedsQL 4.0 scale had four dimensions namely physical, emotional, social, and school functioning making up a total of twenty-three questions [12, 13]. The children’s self-rated version of the scale for children aged 5–18 years old was used in the study. The responses to the questionnaire were scored on the Likert’s scale: ‘0’ for never, ‘1’ for almost never, ‘2’ for sometimes, ‘3’ for often, and ‘4’ for always [10, 11]. The score of each dimension was the average of the sum of the scores of all questions in that dimension. The total score of the questionnaire was the average of the sum of all the questions. The total score of the questionnaire and the total score of each dimension ranged from 0 to 100. The higher the score, the better the QoL.

### Statistical analysis

All analyses were performed using the IBM SPSS software (version 19.0; IBM Corp., Armonk, NY, USA). Statistical significance was set at *p* < 0.05. Continuous variables are presented as mean ± standard deviation (SD) or median (Q25, Q75), while categorical variables are presented as numbers (%). Differences between groups were evaluated using the t-test or Mann–Whitney U test for continuous variables, and the χ2 test for categorical variables. The stepwise multiple linear regression analysis was applied to explore the associations between peri-operative factors and long-term HRQoL scores. In the multiple linear regression model, the right subaxillary approach was set as “1” while the median sternotomy incision approach was set as “0”; parents’ education level was set as “1″, “2″ and” 3″, referring to less than 9 years education, 9–12 years education, and more than 12 years education, respectively; family economic status was set as “1″, “2″ and” 3″, acceding to < local average income, ≈ local average income, and > local average income, respectively; and the rest factors were entered as continuous variables collected peri-operatively.

## Results

### Patient demographics

Between January 2013 and January 2015, a total of 128 patients with VSD combined with PDA underwent closure. Among these patients, 70 were operated on through the right subaxillary approach (group A) and 58 were operated on through median sternotomy incision (group B). The diameter of VSD and PDA in group B was significantly larger than that in group A (6.5 mm vs. 4.2 mm, *p* < 0.01 and 3.9 mm vs. 3.5 mm, *p* < 0.01, respectively). The pulmonary artery pressure in group B was also significantly higher than that in group A (65.0 mmHg vs. 32.0 mmHg, *p* < 0.01) (Table 1).

**Table 1:** Demographic characteristics of patients (group A and group B)

Characteristics	group A (*n* = 70)	group B (*n* = 58)	*p* value
Age (months)	13.0(10.0, 25.0)	2.0(1.0, 3.0)	<.001
Gender			
Male	31	29	0.52
Femal	39	29	0.52
Body weight (kg)	9.0 (8.0, 10.0)	3.0 (3.0, 3.5)	<.001
Classification of VSD			
Perimembranous defect	26	23	0.77
The inlte defect	18	16	0.81
Doubly committed subarterial defect	25	18	0.58
Muscular defect	1	1	0.56
VSD size (mm)	4.2 (3.7, 4.7)	6.5 (5.9,7.3)	<.001
PDA size (mm)	3.5 (3.2, 3.8)	3.9 (3.5, 5.2)	<.001
Pulmonary artery pressure (mmHg)	32.0 (28.0, 35.0)	65.0 (57.5, 73.0)	<.001

*VSD* Ventricular septal defect, *PDA* Patent ductus arteriosus

### Early outcomes

All operations were performed successfully, and no patients from group A needed to be switched to median sternotomy incision. No in-hospital mortality occurred. When comparing intraoperative parameters between group A and group B, the CPB time (58.0 min vs. 72.0 min, *p* < 0.01), aortic cross-clamp time (31.0 min vs. 37.0 min, *p* < 0.01), ventilation time (4.0 h vs. 27.0 h, *p* < 0.01), and hospitalization time (7.0 d vs. 17.0 d, *p* < 0.01) were significantly shorter in group A than in group B (Table 2).

**Table 2:** Intraoperative and postoperative data of patients (group A and group B)

Variables	group A (*n* = 70)	group B (*n* = 58)	*p* value
CPB time (min)	58.0 (50.8, 65.0)	72.0 (68.5, 75.5)	<.001
Aortic cross-clamp time (min)	31.0 (27.0, 35.0)	37.0 (33.0, 42.0)	<.001
Ventilation time (hour)	4.0 (3.0, 6.0)	27.0 (19.0, 32.00	<.001
Hospitalization time (day)	7.0 (6.0, 8.0)	17.0 (13.5，20.0)	<.001
Complications			
Pneumonia	8	11	0.23
Pneumothorax	2	1	0.87
Subcutaneous emphysema	7	1	0.12
Small VSD residual shunt	2	3	0.83
Small PDA residual shunt	1	1	0.56
Mild MR	3	2	0.81
Mild TR	3	3	0.85

*CPB* Cardiopulmonary bypass, *MR* Mitral regurgitation, *TR* Ticuspid regurgitation

The postoperative complications included pneumonia (8 in group A, 11 in group B), pneumothorax (2 in group A, 1 in group B), and subcutaneous emphysema (7 in group A, 1 in group B). There was no significant difference in postoperative complications between the two groups. The postoperative complications are listed in Table 2.

### Follow-up and QoL

The total mean follow-up time was 8.3 ± 1.2 years (7.6 ± 1.6 years in group A and 8.9 ± 2.1 years in group B). Twenty patients were lost to follow-up, and 13 patients withdrew from the study because they could not complete the questionnaire. A total of 95 patients eventually completed the study: 50 (71.4%) in group A, and 45 (82.7%) in group B. All patients were in NYHA class I. There was no breast and pectoral muscle maldevelopment in group A. Five patients in group B had thoracic deformities (3 pectus carinatum and 2 pectus excavatum).

Small residual VSD shunt was detected in 5 patients of whom 3 disappeared on follow-up while 2 were still recorded on ultrasound. Two patients had small PDA residual shunt that had disappeared at follow-up. Mild mitral regurgitation was detected in 5 patients, and mild tricuspid regurgitation was detected in 6 patients. All those with valvular complications remained stable at follow-up.

With regard to HRQoL scores, patients in group A performed better than those in group B in terms of emotional and social functioning dimensions (81.0 vs. 77.0, 81.0 vs. 78.0 *p* < 0.01), as shown in Table 3. In stepwise multiple linear regression analysis, the right subaxillary approach (B 3.56; 95% confidence interval [CI] 1.65–5.46; *p* < 0.01), higher parents’ education level (B 1.62; 95% CI 0.65–2.31; *p* < 0.01), and better family economic status (B 1.48; 95% CI 0.79–2.45; *p* < 0.01) were related to the HRQoL scores (Table 4).

**Table 3:** Health-related quality of life scores in group A and group B during follow-up

Variables	group A (*n* = 50)	group B (*n* = 45)	*p* value
Parents’ education level			
< High school (n, %)	8 (16.0%)	5 (11.1%)	0.49
High school (n, %)	24 (48.0%)	23 (51.1%)	0.76
College or beyond (n, %)	18 (36.0%)	17 (37.8%)	0.86
Family economic status			
Poor (n, %)	5 (10.0%)	5 (11.1%)	0.86
Average (n, %)	26 (52.0%)	21 (46.7%)	0.60
Rich (n, %)	19 (38.0%)	19 (42.2%)	0.68
HRQoL domain			
Physical functioning	83.0 (81.0, 84.0)	82.0 (80.0, 83.0)	0.12
Emotional functioning	81.0 (80.0, 82.0)	77.0 (76.0, 78.0)	<.001
Social functioning	81.0 (79.0, 82.0)	78.0 (77.0, 79.0)	<.001
Shchool functioning	81.8 (81.8, 83.0)	81.0 (80.0, 82.0)	0.23
Total score	81.0 (80.0, 82.3)	80.0 (79.0, 81.0)	0.31

*HRQoL* Health-related quality of life

**Table 4:** The factors related to health-related quality of life scores for all patients during follow-up

Variables	B	95% Confidence Interval		*P* value
		Lower Bound	Upper Bound	
Right subaxillary approach	3.56	1.65	5.46	< 0.01
Age	−0.01	−0.06	0.05	0.95
Gender	0.23	−0.24	0.70	0.34
Body weight	−0.01	− 0.19	0.18	0.94
VSD size	−0.20	−0.47	0.08	0.16
PDA size	0.01	−0.42	0.43	0.98
Pulmonary artery pressure	0.05	0.01	0.09	0.12
CPB time	0.01	−0.03	0.41	0.75
Aortic cross-clamp time	−0.03	−0.08	0.02	0.18
Ventilation time	−0.01	−0.06	0.03	0.54
Hospitalization time	0.07	−0.24	0.69	0.34
Higher parents’ education level	1.62	0.65	2.31	< 0.01
Better family economic status	1.48	0.79	2.45	< 0.01

*VSD* Ventricular septal defect, *PDA* Patent ductus arteriosus, *CPB* Cardiopulmonary bypass

## Discussion

Median incision had been the standard surgical approach for the correction of CHD [6]. However, due to obvious surgical scars and thoracic deformity, patients have become less accepting of this surgical technique [6]. The right subaxillary incision has been widely used in the repair of simple CHD due to its cosmetic benefits.

In this study, the patients with VSD and PDA who underwent closure through the right subaxillary approach had good early outcomes. The CPB time, aortic cross-clamp time, ventilation time, and hospitalization time of the right subaxillary approach group were shorter than those of the median sternotomy group. It was possible that because the patients in the median sternotomy group were younger, had poorer tolerance to surgical distress, and had lower weight than those in the right subaxillary approach group, it took them longer to recover. Shi et al. reported that children aged ≤3 months required prolonged mechanical ventilation after cardiac surgery [14]. In addition, Du et al. suggested that age was one of the risk factors for low cardiac output syndrome in children with CHD undergoing cardiac surgery [15]. In the current study, children in the median sternotomy group were all young and had low weight, the large diameter of VSD and PDA, high pulmonary artery pressure, and were prone to respiratory and cardiac insufficiency. Although these factors might affect the ventilation time and hospitalization time of patients, the operation was successful in both groups, with only minor complications and overall satisfactory clinical results.

Our study showed that the mid-term follow-up results of children with VSD and PDA were excellent. The intracardiac malformations were satisfactorily corrected, and the patients were all in NYHA class I. There was no breast and pectoral muscle maldevelopment in the subaxillary approach group. However, five patients in the median sternotomy group had thoracic deformities. Many minimally invasive incisions, such as mini-sternotomy, and anterolateral and posterolateral thoracotomies, were used in repairing common CHD [16–18]. However, some patients who underwent procedures through these approaches would have some long-term complications, such as thoracic deformity, and breast and pectoral muscle dysplasia [18]. However, the right subaxillary approach was far away from the breast and pectoral muscle, which entered into the thoracic cavity through the intercostal space. Because the incision was hidden and did not affect the growth of breast tissue and pectoral muscle, an increasing number of cardiac surgeons were choosing the right axillary incision to repair common CHD [19].

The right subaxillary incision had been widely used in the correction of simple CHD. However, only a few studies had reported the analysis of the postoperative QoL of children of school age during follow-up [20, 21]. According to our results, based on the HRQoL scores, children who underwent the right subaxillary approach performed better in terms of emotional and social functioning dimensions than those who underwent median sternotomy. The right subaxillary approach had concealed incisions and could completely cover the scars when the arms were sagging. In contrast, the children with median incisions had obvious scars and even thoracic deformities (e.g., pectus carinatum and pectus excavatum), which were associated with negative emotions, such as inferiority complex, depression, and impairment in social activities [20].

The current study also showed that the right subaxillary approach, higher parents’ education level, and better family economic status were associated with higher HRQoL scores. As we all know, the perfect corporeal image played an important role in promoting positive self-esteem. A visible median sternal surgical scar, or even a thoracic deformity, was a lifelong reminder of heart problems, which could cause negative social and psychological consequences [22]. Eslami and colleagues reported that annual income is associated with the QoL of adults with CHD [23]. Im and colleagues reported the New York Heart Associated class, presence of siblings, and mother’s emotional warmth are related to the QoL of patients with CHD [21], which was different from our results. This might be related to the education level and economic income of citizens in different countries. Wang and colleagues showed that a good knowledge of their cardiac condition, optimism, and adequate social support had a positive impact on the QoL for adolescents with heart disease [24]. In conclusion, there were many factors affecting the QoL of patients with CHD. These children should receive more active psychological intervention and social support.

### Limitations

This was a retrospective study with a small sample size. There was selection bias for the two surgical approaches; however, a randomized controlled trial design would have been difficult to conduct and would not be ethically approved. In stepwise multiple linear regression analysis, age and weight were not associated with the QoL of patients. Therefore, this selection bias might have no significant effect on the QoL scores.

## Conclusions

In selected patients, the right subaxillary approach was a feasible and effective technique for VSD with PDA. Younger and smaller patients who were operated on using median sternotomy incisions due to large defects and pulmonary hypertension had lower QoL scores. The right subaxillary approach, higher parents’ education level, and better family economic status were related to higher QoL scores.

## Data Availability

All data generated or analysed during this study are included in this published article.
